# Phenotypic Plasticity and Cell Fate Decisions in Cancer: Insights from Dynamical Systems Theory

**DOI:** 10.3390/cancers9070070

**Published:** 2017-06-22

**Authors:** Dongya Jia, Mohit Kumar Jolly, Prakash Kulkarni, Herbert Levine

**Affiliations:** 1 Center for Theoretical Biological Physics, Rice University, Houston, TX 77005, USA; dyajia@gmail.com (D.J.); mkjolly.15@gmail.com (M.K.J.); 2 Graduate Program in Systems, Synthetic and Physical Biology, Rice University, Houston, TX 77005, USA; 3 Institute for Bioscience and Biotechnology Research, University of Maryland, Rockville, MD 20850, USA; pkulkar4@ibbr.umd.edu; 4 Department of Bioengineering, Rice University, Houston, TX 77005, USA; 5 Department of Physics and Astronomy, Rice University, Houston, TX 77005, USA; 6 Department of Biosciences, Rice University, Houston, TX 77005, USA

**Keywords:** cell fate decision, cancer attractors, gene network dynamics, EMT, therapy resistance, intrinsically disordered proteins

## Abstract

Waddington’s epigenetic landscape, a famous metaphor in developmental biology, depicts how a stem cell progresses from an undifferentiated phenotype to a differentiated one. The concept of “landscape” in the context of dynamical systems theory represents a high-dimensional space, in which each cell phenotype is considered as an “attractor” that is determined by interactions between multiple molecular players, and is buffered against environmental fluctuations. In addition, biological noise is thought to play an important role during these cell-fate decisions and in fact controls transitions between different phenotypes. Here, we discuss the phenotypic transitions in cancer from a dynamical systems perspective and invoke the concept of “cancer attractors”—hidden stable states of the underlying regulatory network that are not occupied by normal cells. Phenotypic transitions in cancer occur at varying levels depending on the context. Using epithelial-to-mesenchymal transition (EMT), cancer stem-like properties, metabolic reprogramming and the emergence of therapy resistance as examples, we illustrate how phenotypic plasticity in cancer cells enables them to acquire hybrid phenotypes (such as hybrid epithelial/mesenchymal and hybrid metabolic phenotypes) that tend to be more aggressive and notoriously resilient to therapies such as chemotherapy and androgen-deprivation therapy. Furthermore, we highlight multiple factors that may give rise to phenotypic plasticity in cancer cells, such as (a) multi-stability or oscillatory behaviors governed by underlying regulatory networks involved in cell-fate decisions in cancer cells, and (b) network rewiring due to conformational dynamics of intrinsically disordered proteins (IDPs) that are highly enriched in cancer cells. We conclude by discussing why a therapeutic approach that promotes “recanalization”, i.e., the exit from “cancer attractors” and re-entry into “normal attractors”, is more likely to succeed rather than a conventional approach that targets individual molecules/pathways.

## 1. Introduction

“The woods are lovely, dark and deep, but I have promises to keep, and miles to go before I sleep, and miles to go before I sleep.”

—*Robert Frost*

**Figure 1:**
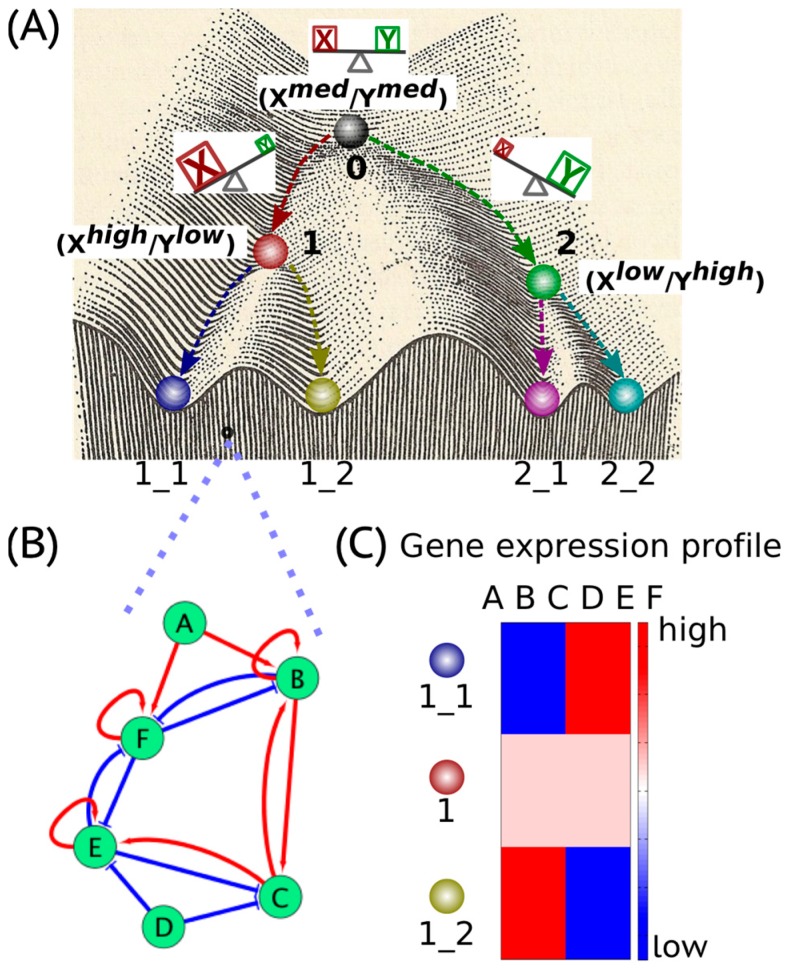
Schematic illustration of Waddington’s epigenetic landscape. (**A**) Waddington’s epigenetic landscape (adopted and revised from [1]). The balls with different colors on the landscape represent different cell phenotypes, each settles steadily in one of the sub-valleys at the foot of the hill. X and Y are the master regulators driving a cell to attain the phenotypes “1” and “2” respectively. The phenotype “0”, characterized by the co-expression of both X and Y at a medium level X^*med*^/Y^*med*^, represents the progenitor state of the two differentiated states “1” and “2” which are characterized by X^*high*^/Y^*low*^ and X^*low*^/Y^*high*^ respectively. Due to inherent stochasticity in the progenitor cell “0”, the level of X (Y) becomes higher than that of Y (X). This asymmetry can trigger a cascade of events where the levels of X (Y) continually increase and those of Y (X) continually decrease, because X (Y) can progressively repress its repressor Y (X) strongly, rendering its own inhibition by Y (X) ineffective. Consequently, the cell attains the differentiated state X^*high*^/Y^*low*^ (X^*low*^/Y^*high*^). (**B**) Schematic illustration of a gene regulatory network (GRN) governing the differentiation of “1” to two lineages “1_1” and “1_2”. The nodes A–F represent different genes whose regulatory behaviors usually can be approximated by the interplay between two master regulators X and Y as aforementioned. Various kinds of regulation can be found in the GRN, such as transcriptional activation, represented by red arrows, transcriptional inhibition, represented by blue bar-headed arrows, and self-activation, represented by circled arrows. (**C**) Schematic illustration of a heatmap that depicts the gene expression patterns of different cell phenotypes. The two sister lineages “1_1” and “1_2” are characterized by different gene expression patterns, i.e., relatively high expression of one gene set and low of another. The progenitor of “1_1” and “1_2”, i.e., “1”, usually co-expresses both sets of genes at some intermediate level.

Waddington’s epigenetic landscape [1] depicting how a stem cell progresses from an undifferentiated phenotype to a differentiated one is one of the most famous and powerful metaphors in developmental biology. Conceptually, the differentiation of a stem cell is represented by a ball rolling downhill through a rugged landscape of bifurcating valleys, each representing a possible cell fate (Figure 1A). The valleys continue bifurcating and the ball finally enters one of many sub-valleys at the foot of the hill. These sub-valleys represent terminally differentiated states, i.e., cell fates. The cell is held permanently, unless perturbed significantly, in the terminally differentiated state by high ridges, i.e., valley walls. The deeper the valley, the more canalized the cell fate. The epigenetic landscape in the context of dynamical systems theory represents a high-dimensional state space in which each cell fate is an “attractor” shaped by the architecture of its regulatory interaction network [2]. It is generally held that cell fate is essentially irreversible; it follows the “arrow of time”. However, recent developments in cellular reprogramming have illustrated that a terminally differentiated cell can be forced to switch states (phenotypes) and acquire an undifferentiated state by supraphysiological overexpression of a cocktail of transcription factors (TFs) [3]. Similarly, cancer has been also shown to be ‘reversed’ to a non-malignant phenotype, thereby raising questions about the sufficient and necessary role of mutations in cancer progression [4].

In a dynamical system, an “attractor” (steady state) represents a set of values of the variables towards which the system evolves from a wide variety of starting conditions, and is robust to slight perturbations. Cell phenotypes are regulated by underlying gene regulatory networks (GRNs) (Figure 1B). GRNs are dynamical systems that start from context-dependent conditions, develop temporally due to the mutual interactions between molecular regulators (genes, proteins, microRNAs etc.) and later settle down into “attractors” (stable cell phenotype), each of which is characterized by a unique gene expression pattern (Figure 1C). Different possible steady states (“attractors”) of a given GRN can be identified by mathematically modeling its dynamics; each attractor is associated with a steady-state probability of finding the system in that particular configuration. Together, this set of attractors—with their relative probabilities of being realized by the system—define a “landscape”. Representing a stable cell phenotype as an “attractor” has helped realize the basic concepts of both single-cell stochasticity and population determinism, i.e., single cells can shift from one attractor to another due to noise, without altering the overall population structure. This perspective facilitates viewing biological systems from the perspective of statistical mechanics, where a macrostate (a cell population structure) can correspond to multiple microstates (phenotypic heterogeneity at a single-cell level) [5].

The concept of an “attractor” representing a cell phenotype (cell fate) has been widely used to understand lineage specifications during development. Usually, lineage commitment between sister cell-fates (i.e., sharing a common progenitor) is a binary branching process that is governed by a decision-making circuit consisting of two transcription factors X and Y that mutually inhibit each other and can also self-activate [2], referred to as a “self-activating toggle switch” [6]. X and Y are usually the master regulators of the two sister cell-fates. Such a “self-activating toggle switch” usually generates three stable “attractors’ that are characterized by X^*high*^/Y^*low*^, X^*low*^/Y^*high*^ and X^*medium*^/Y^*medium*^ corresponding to two differentiated cell fates and an undifferentiated progenitor state respectively [2,6,7] (Figure 1A). Such “self-activating toggle switches” governing lineage commitments have been studied in various scenarios, such as the Gata1/PU.1 switch in the lineage commitment of multipotent progenitor cells [8], the Cdx2/Oct4 switch in the differentiation of a totipotent embryo [9], the Gata6/Nanog switch in the branching process of inner cell mass [10] and the T-bet/Gata3 switch in the lineage specification of the T-helper cells [11].

The concept of an “attractor” representing a cell phenotype is used not only in understanding embryonic development, but also in elucidating cancer initiation and progression. Cancer cells are regarded as abnormal cell phenotypes, i.e., “cancer attractors”, and are believed to be the “hidden stable states” enabled by the regulatory networks that are not commonly occupied by normal cells [10]. Accesses to “cancer attractors” can be facilitated by genetic events (mutations) and/or non-genetic events (contextual signals and biological noise). For example, loss-of-function mutations in tumor suppressor genes such as TP53 and BRCA and/or gain-of-function mutations in proto-oncogenes such as MYC and RAS facilitate oncogenic properties of cells [12]. In addition to genetic events, the microenvironment surrounding cells can also promote tumorigenesis. For instance, overexpression of a stromal proteinase-matrix metalloproteinase-3 (MMP3) in both mouse phenotypically normal mammary epithelial cells (Scp2) and the mammary glands of transgenic mice, results in a reactive stroma and eventually leads to infiltrative mammary tumors [13]. Similarly, overexpression of the platelet-derived growth factor subunit B (PDGF-B) in the non-tumorigenic immortalized human keratinocytes (HaCaT) leads to a conversion to epithelial tumor cells through stromal cell activation [14]. These examples suggest that the probability to get access to “cancer attractors” can be enhanced due to gene mutations and/or contextual signals in the microenvironment. Furthermore, transitions can happen among “cancer attractors” to benefit cancer cells for survival and progression, referred to as phenotypic plasticity in cancer [15].

In this review, we invoke the concept of “cancer attractors” and discuss the phenotypic plasticity of cancer cells from a dynamical systems perspective. Using epithelial-to-mesenchymal transition (EMT) and the acquisition of stem-like properties, metabolic reprogramming and the emergence of drug/hormone resistance in cancer as examples, we illustrate how non-genetic heterogeneity regulates phenotypic plasticity of cancer cells that enables them to acquire phenotypes that are notoriously aggressive and resilient to drug/hormone treatment. With enhanced plasticity, cancer cells can potentially rewire the regulatory network to access latent “attractors” suggesting that cancer initiation and progression may, at least in part, be due to a “de-canalization” of normal cell fates. Finally, we highlight the potential role of intrinsically disordered proteins (IDPs) that comprise a vast majority of the proteins over-expressed in cancer, and how biological noise due to IDP conformational dynamics may further enhance phenotypic plasticity of cancer cells. Since the perspective is intended to encourage cross pollination of ideas between biologists, especially cancer biologists, and physicists interested in exploring the physics of biology, technical jargon is limited to its minimum and equations are omitted.

## 2. Cancer Cell States: The Hidden “Attractors”

Cell phenotypes manifested during embryonic development are governed by specific gene regulatory networks (GRNs) (Figure 1B). The GRNs give rise to an epigenetic landscape consisting of multiple stable gene expression patterns (Figure 1C) characterizing various “attractors”, i.e., “stable states” or “phenotypes” [16,17]. The “attractors” are usually self-stabilized and robust to local perturbations [18]. However, certain transitions between “attractors”, i.e., phenotypic switching, can be triggered by regulatory signals, such as cytokines and noise due to gene expression as well as IDP conformational dynamics in addition to mutational events [19,20].

Cancer cells are viewed as abnormal cell types that are characterized by hallmarks such as sustained proliferation, invasion and metabolic reprogramming [21]. Extensive inherent heterogeneity of cancer cells has been shown at both the genetic level due to genomic instability [22], and the non-genetic level, resulting from cellular plasticity, i.e., the ability of cells to switch between phenotypes [23,24]. The examples of non-genetic heterogeneity in cancer include, but are not restricted to, epithelial-to-mesenchymal transition (EMT) [25], acquiring “stem-like” properties [26], and metabolic plasticity [27,28]. In certain cases, these processes have been shown to be coupled. For instance, cells undergoing EMT can acquire stem-like properties [29], stem-like properties associate with metabolic changes [30], and metabolic programming involves changes in EMT [27,31,32].

This extensive plasticity of cancer cells may enable the occupancy of the “attractors” that are unpopulated or inaccessible during embryonic development, or equivalently, acquire phenotypes not usually observed during development or homeostasis. The concept of “cancer attractors” representing abnormal cell types was first proposed by Stuart Kauffman in 1971 [33] and recently revisited by Huang, Ao and colleagues [34,35]. In the following sections, we will review progress in elucidating the phenotypic plasticity of cancer cells from the dynamical systems perspective, namely, by viewing cancer cell phenotypes as different “cancer attractors” in the state space determined by the underlying regulatory networks.

## 3. Cell Fate Decision-Making during Epithelial-to-Mesenchymal Transition

Epithelial-to-Mesenchymal Transition (EMT) is a trans-differentiation program by which epithelial cells lose their cell-cell adhesion and gain migratory property to become mesenchymal cells. Both EMT and its reverse—Mesenchymal-to-Epithelial Transition (MET)—play crucial roles during embryogenesis (during processes such as gastrulation, neural crest delamination and myogenesis) and tissue repair (during wound healing and fibrosis) [36]. However, EMT may sometimes be “hijacked” by carcinoma cells to acquire enhanced migratory properties that can contribute to metastasis and/or acquired therapy resistance [37,38]. Moreover, the EMT transcription factors (EMT-TFs), such as ZEB (zinc finger E-box-binding homeobox) and SNAIL (zinc finger protein SNAI1), have even been shown to play an important role in tumor progression in non-carcinomas, such as melanoma [39,40] and glioblastoma [41,42].

**Figure 2:**
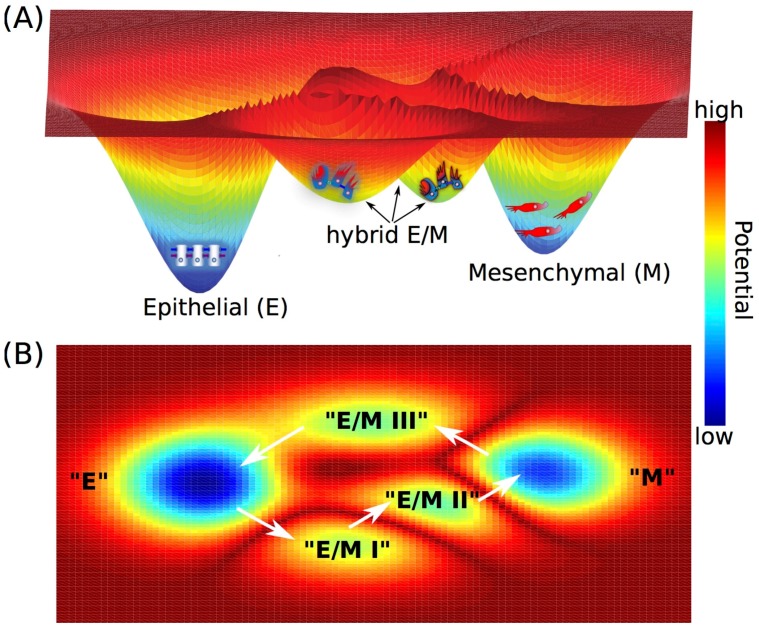
Schematic illustration of the quasi-potential landscape for epithelial-to-mesenchymal transition (EMT) in 3-dimensional space (**A**) and 2-dimensional projection (**B**). In (**A**), the basins of attraction depicting the attractors “E”, “E/M” and “M” are labeled respectively along with the cartoons representing the epithelial (tight cell-cell adhesion, cobblestone shaped), hybrid E/M (some cell-cell adhesion and invasive) and mesenchymal (no cell-cell adhesion, invasive and spindle-shaped) phenotypes. The quasi-potential of “attractors”, i.e., stability of “attractors”, is derived from the probability of finding cells in that “attractors”. Lower potential here represents more stable “attractor” in the landscape. The “potential well” depicted here is an analog of “valleys” in Waddington epigenetic landscape.

During metastasis, cancer cells do not always undergo a complete EMT, instead a partial EMT (leading to a hybrid epithelial/mesenchymal (E/M)) phenotype, in which cells exhibit both epithelial (cell-cell adhesion) and mesenchymal (migration and/or invasion) traits, has often been observed [43,44,45] (Figure 2A). Cells in a hybrid E/M phenotype can migrate collectively as a cluster instead of migrating individually like a cell that has undergone a complete EMT. These clusters of circulating tumor cells (CTCs) associate with up to 50-fold higher metastasis potential and higher tumor-initiating potential compared with single CTCs [43,46], thus being proposed as the primary “bad actors” of metastasis [44].

To understand the epithelial-mesenchymal plasticity, i.e., transitions among epithelial (E), hybrid E/M and mesenchymal (M) phenotypes, a core EMT regulatory circuit consisting of two transcription factor families—ZEB and SNAIL and two microRNA families—miR-200 and miR-34, has been characterized. High expression of the transcription factors ZEB and SNAIL promotes a mesenchymal phenotype while high expression of microRNAs miR-200 and miR-34 maintains an epithelial phenotype. Two mathematical models [47,48,49] that were independently proposed have been applied to analyze the dynamics of the core EMT circuit. Both models elucidate that (1) the core EMT decision-making circuit functions as a “three-way” switch, that can give rise to three stable states—“E” characterized by (E marker^*high*^/M marker^*low*^), “M” characterized by (E marker^*low*^/M marker^*high*^) and “E/M” characterized by (E marker^*medium*^/M marker^*medium*^). (2) EMT is a two-step processes—from “E” to “E/M” to “M” [47,48]. Once the cells transition into a mesenchymal phenotype, the stable state or phenotype “M” can be self-stabilized, by feedback loops such as increased inhibition of ZEB on miR-34 [50], and/or the decreased inhibition of miR-200 on the endogenous TGF-β [48,50]. The landscape approach has been utilized to quantify the transition processes among these three stable states, i.e., “attractors”—“E”, “E/M” and “M” [51]. This study suggested that attainment of a hybrid E/M state often decreases the required strength of EMT-inducing signals to initiate EMT, i.e., pulling cells out of the stable state “E”, thus enabling cancer cells to be more plastic [51].

The hybrid E/M phenotype has been observed in circulating tumor cells (CTCs), primary tumors, metastases, and 3D reconstructions of 2D histological sections [44,52], but it has tacitly been largely assumed as a “metastable” or transient phenotype [53]. However, recently, in part driven by these mathematical models, a stable hybrid E/M phenotype has been observed in the non-small cell lung cancer (NSCLC) cell line−H1975, in which individual cells co-express an epithelial marker—E-cadherin and a mesenchymal marker−Vimentin [54]. These cells can maintain their hybrid E/M phenotype for over two months after multiple passages, thus being characterized as a stable phenotype [54]. Moreover, such an integrated computational-experimental analysis has also helped identify two transcription factors GRHL2 (grainyhead like transcription factor 2) and OVOL2 (ovo-like zinc finger 2) that can stabilize the hybrid E/M phenotype [54,55,56]. Knockdown of either GRHL2 or OVOL2 in H1975 cells destabilized the hybrid E/M phenotype and cells progressing to a complete EMT state [54]. Thus, these “phenotypic stability factors” (PSFs) GRHL2 and OVOL [57] act as “critical molecular brakes” by preventing “cells that have gained partial plasticity from crossing the line to undergo complete EMT” [58]. Of note, there may exist multiple hybrid E/M phenotypes characterized by different gene expression profiles [56,59], and other players such as JAG1 (ligand of cell-cell communication pathway—Notch signaling) and ∆NP63α can also act as PSFs [60,61]. EMT and MET need not be symmetric [47], i.e., EMT and MET could potentially proceed via different hybrid E/M phenotypes, that enables cancer cells to have more phenotypic plasticity (Figure 2).

## 4. EMT and Stemness

Cancer cells undergoing EMT can acquire stemness, i.e., stem-like properties or tumor-initiation potential [29], and thus behave operationally as Cancer Stem Cells (CSCs) as observed in multiple solid tumors [62]. The coupling between EMT and stemness is finely regulated. On the one hand, EMT promotes the acquirement of stemness in breast [29,63] hepatocellular [64], pancreatic [65] and colorectal [66] carcinomas; on the other hand, repression of EMT is required for tumor initiation and metastatic colonization [67,68,69].

As the first step to understand the coupled decision-making of EMT and stemness, Jolly et al. [70] formulated a mathematical model to analyze the dynamics of the coupled decision-making circuits of EMT-ZEB/miR-200 and stemness-LIN28/let-7 [71]. It suggests that the “stemness window” is most likely to lie at an intermediate position on the “EMT axis” with E and M phenotypes as the two ends. Further, this positioning of “stemness window” can be adjusted and the phenotypic stability factors such as OVOL promote the association of a hybrid E/M phenotype with stemness, a prediction that has been supported by recent experimental work. For instance, HMLER breast cancer cells co-expressing both epithelial and mesenchymal genes, thus being characterized as hybrid E/M cells, exhibited highest mammosphere formation potential compared with epithelial and mesenchymal HMLER cells [72]. Besides, Cancer Stem Cell (CSC)-enriched population resides in a hybrid E/M phenotype of triple-negative breast cancer cells [73]. Last but not least, a subpopulation of normal mammary cells, accompanied by both epithelial-like and mesenchymal-like characteristics, i.e., hybrid E/M phenotype, displays the highest mammosphere-formation capacity [74]. Thus, a biphasic relationship between stemness and EMT—stemness increases initially during EMT progression, but then subsides as cells complete EMT—seems to be the emerging notion [43,75,76].

CSCs have also been observed to display enriched drug resistance [77]. For example, a hybrid E/M phenotype has been reported to be resistant to paclitaxel and salinomycin [78]. Moreover, adaptive drug resistance involves transitioning to a CD24^*high*^CD44^*high*^ state [79]—a proposed signature for hybrid E/M phenotype [72]. Future work on quantifying the landscape [80] for the coupled circuits of EMT and stemness, along with a better mechanistic understanding of drug resistance pathways, are required to generate valuable insights into the EMT-stemness interplay.

## 5. Metabolic Reprogramming and EMT

Abnormal metabolism is an emerging hallmark of cancer [21,81]. Unlike normal cells, cancer cells mainly utilize glycolysis for ATP production even in presence of oxygen, a phenomenon referred to as aerobic glycolysis or the Warburg effect [82]. Although aerobic glycolysis has been proposed to be the dominant metabolism phenotype in cancer cells [83,84], emerging evidence shows that mitochondria in cancer cells are actively functioning and oxidative phosphorylation (OXPHOS) can enhance metastasis [85,86,87,88,89,90].

As the first step to understand the metabolic plasticity in cancer, Yu et al. [91] constructed a core metabolism regulatory network consisting of AMPK and HIF-1—master regulators for OXPHOS and glycolysis, respectively—and ROS (reactive oxygen species) that mediates the interplay between AMPK and HIF-1. This AMPK:HIF-1:ROS regulatory network enables three stable states—(pAMPK^*high*^/HIF-1^*low*^), (pAMPK^*low*^/HIF-1^*high*^) and (pAMPK^*medium*^/HIF-1^*medium*^)—corresponding to an OXPHOS, a glycolysis and a hybrid OXPHOS/glycolysis metabolic phenotype respectively (pAMPK denotes phosphorylated AMPK, i.e., the active form of AMPK). The hybrid metabolic state, in which cancer cells can utilize both glycolysis and OXPHOS, facilitates relatively high plasticity for ATP production and proliferation for cancer cells. The hybrid metabolism phenotype can be stabilized by increased HIF-1 activity, high oncogene (MYC, RAS, c-SRC) activity and high mitochondria ROS production in cancer cells compared with that in normal cells [91].

The hybrid metabolism phenotype proposed by the aforementioned modeling work has been observed in many experimental studies to be associated with metastatic potential. The supermetastatic human tumor cells SiHa-F3 by in vitro selection and the mouse melanoma cells B16F10, B16-M1 to M5 by in vivo selection have an increased OXPHOS activity together with an enhanced invasive activity [92]. The non-small cell lung carcinoma A549 cells undergoing EMT induced by TGF-β show elevated respiration [27]. The metastatic breast cancer cells 66cl4 and 4T1 have both enhanced oxidative as well as glycolytic metabolism accompanied by increased extracellular acidification rate and oxygen consumption rate compared with non-metastatic 67NR cells [93]. In addition, cells in the hybrid metabolism phenotype can maintain ROS at a moderate level [91], thus avoiding excessive DNA damage [94] while using ROS signaling to promote metastasis [95]. Moreover, cells in the hybrid phenotype can simultaneously produce energy and generate biomass for proliferation [30]. Therefore, a combination therapy that target the hybrid metabolism phenotype, i.e., blocking both glycolysis and OXPHOS in cancer cells, could be relatively more effective [30,91] than the therapy targeting only one metabolic pathway.

Of note, regulation of metabolic plasticity has been shown to be coupled with the EMT decision-making [31]. EMT enhances glycolysis in MCF-7 and BT-474 cells [96] while shifts metabolism from glycolysis to OXPHOS in MCF10 cells [97]. Fatty acid oxidation is more utilized in the mesenchymal breast cancer cells D492M than that in epithelial cells D492 (D492M cells are isolated following a spontaneous EMT in D492 cells) [32]. Blocking fatty acid oxidation in MDA-MB-231 cells decreases their migratory and colony-formation properties, suggesting multiple feedback loops between regulatory circuit of metabolism, EMT and stemness [90]. This situation remains to be clarified on the basis of models.

Metabolic plasticity has also been observed in CSCs. Epithelial-like CSCs, characterized by ALDH^*high*^, have higher oxygen consumption rate and lower glycolytic activity compared with the mesenchymal-like breast CSCs, characterized by CD44^*high*^CD24^*low*^ [98,99]. Recent work highlighted that ALDH^*high*^ cells may exhibit a hybrid E/M state [74]. Future work to analyze the coupled decision-making of metabolism, EMT and stemness needs to be done to comprehensively chart the stable states characterized by varied EMT, stem-like property and metabolism traits, while taking into consideration the direct coupling between gene expression and metabolites, at least partly through epigenetic mechanisms [100].

## 6. EMT and Therapy Resistance

EMT has been associated with both *de novo* and acquired resistance. *De novo* resistance implies intrinsic refractory response of patients, whereas acquired resistance refers to cases where patients first respond to therapy but later relapse. A relationship between EMT and *de novo* resistance has been well studied in cases of targeted therapy. For instance, increased levels of E-cadherin were associated with sensitivity to EGFR kinase inhibitors such as gefitinib in non-small-cell lung cancer (NSCLC) cell lines, and pre-treatment of resistant cell lines to induce E-cadherin levels improved their sensitivity [101]. Similarly, knockdown of the levels of SLUG, an EMT-TF, in *de novo* trastuzumab-resistant HER2+ breast cancer cells can drive them to being sensitive to trastuzumab [102]. Besides, recent in vivo reports that questioned an indispensable role of EMT in metastasis only strengthened a potential causal role of EMT in driving chemoresistance. For example, knocking down TWIST or SNAIL sensitized tumors to gemcitabine in pancreatic cancer mouse models [103], and miR-200 overexpression abrogated resistance to cyclophosphamide, a drug commonly used in breast cancer [104]. Taken together, these studies suggest that cellular plasticity mediated by EMT can act as a switch enabling cells to “enter” and “exit” a drug-resistant cell state dynamically. Recent mathematical modeling attempts that investigate the crosstalk among signaling players have highlighted that non-genetic heterogeneity can drive this dynamic “entry” into and “exit” from a stem-like therapy-resistant state [70,71,80,105,106].

This dynamic “entry” and “exit” may also underlie acquired or adaptive drug resistance, where different therapies may induce cells to access the “cancer attractors” which are relatively inaccessible otherwise, but can be used to play “hide-and-seek” with different therapies. For instance, in ovarian cancer, treatment with chemotherapeutic drugs such as cisplatin, doxorubicin, and paclitaxel can reversibly increase a small population of CXCR4^*high*^ cells that is drug-resistant, mesenchymal-like, and has enhanced tumor-initiation potential [107]. Other examples of adaptive resistance include melanoma cells switching to a NGFR^*high*^ state upon exposure to RAF/MEK inhibitors [108], NSCLC cells upregulating ZEB1 on prolonged exposure to increasing concentrations of erlotinib [109], vemurafenib driving epigenetic reprogramming to a drug-resistant state in melanoma [110] and chemotherapy enriching a CD24^*high*^CD4^*high*^ drug-resistant population in breast cancer cells [79].

Mechanism-based mathematical models have helped tease out that this adaptive enrichment of a drug-resistant cancer subpopulation can result from phenotypic plasticity, for instance, the emergence of a drug-resistant CD24^*high*^/CD44^*high*^ state [79]. The CD24^*high*^/CD44^*high*^ state was also suggested to associate with an elevated Notch-Jagged signaling, a prediction that has been validated experimentally at least preliminarily [60]. Similarly, in an attempt to understand the experimentally observed correlation between EMT and immune evasion, a mathematical model involving the transcription factors STAT1, STAT3, and the microRNA miR-200 predicted and guided the experimental design for how inhibiting STAT3 activation altered the levels of a set of immune-evasion mediators PSMB8 and PSMB9 in the mesenchymal NSCLC cells [111]. Therefore, mathematical models can be valuable tools in elucidating the principles of phenotypic plasticity governing both *de novo* and acquired resistance to various therapies.

## 7. Role of Intrinsically Disordered Proteins in Phenotypic Plasticity

From the foregoing, it is obvious that cancer cells retain high plasticity which facilitates phenotypic transitions among various phenotypes to adjust to microenvironments. A hallmark of many master regulators that regulate cancer phenotypic plasticity such as, oncoproteins that cause cellular transformation, factors that induce reprograming of somatic cells to pluripotent stem (iPS) cells, and several EMT-TFs that play a critical role in EMT/MET is that, they are intrinsically disordered proteins (IDPs) [20,112,113,114].

IDPs are proteins, or large regions within ordered proteins, that lack three-dimensional structure. They exist as ensembles instead but can transition from disorder to order upon interacting with a biological target (reviewed in [115,116]). However, there are several cases where IDPs stochastically sample the conformational state space *a priori* [117,118] or are functional even when remaining highly disordered [119,120,121,122]. Regardless however, because IDPs populate multiple conformational states albeit transiently, and display rapid conformational dynamics, they are prone to stochastically engage in myriad “promiscuous” interactions, especially when they are overexpressed [123,124].

In an attempt to understand the roles of IDPs in cancer phenotypic plasticity, Mahmoudabadi et al. [125] have suggested that these promiscuous interactions result in “noise” in the system. Further, to distinguish this noise from the widely recognized “transcriptional noise” that stems from gene expression, the authors coined the term “conformational noise”. This new source of biological noise stems from IDP conformational dynamics and is an inherent characteristic of IDP interactions. However, notwithstanding the distinction, the authors postulated that just like transcriptional noise which plays an important role in generating phenotypic heterogeneity [126,127], the collective effect of conformational noise is an ensemble of protein regulatory network configurations, from which the most suitable configuration can be explored by the cancer cell to “make” appropriate decisions, thus conferring it with remarkable phenotypic plasticity. Moreover, the ubiquitous presence of intrinsic disorder in transcriptional factors and, more generally, in proteins that occupy hub positions in regulatory networks is thought to be indicative of the role of IDPs in propagation and amplification of transcriptional as well as other types of noise (e.g., noise in signaling pathways) in the system. Therefore, as effectors of conformational and transcriptional noise, IDPs can rewire regulatory networks unmasking latent regulatory circuits in response to perturbations and switch phenotypes to generate phenotypic heterogeneity [125]. Thus, from Waddington’s epigenetic landscape perspective, conformational noise-driven rewiring results in the system exploring the high-dimensional state space and homing to attractor basins that harbor “cancer attractors”. Implicit in the model proposed by Mahmoudabadi et al. [125], phenotypic switching can result from stochastic (non-genetic) rather than by deterministic events alone (genetic), and the regulatory network configuration contains information that can aid cell fate decisions.

In a recent paper, Mooney et al. [20] reviewed the role of IDPs in EMT and discussed how IDP conformational dynamics can contribute to phenotypic plasticity using prostate cancer (PCa) as an example. In addition, Kulkarni et al. [106] discussed the role of IDPs in the emergence of androgen resistance (independence), yet another paradigm of phenotypic plasticity in PCa. Here, we highlight their role in the emergence of androgen resistance.

The onset of androgen resistance in patients treated with androgen-deprivation therapy (ADT) is a major impediment in PCa. However, the underlying molecular mechanisms are not fully understood. To gain new insight, Kulkarni et al. [106] recently employed multiple biophysical approaches that report conformational preferences of Prostate-Associated Gene 4 (PAGE4). PAGE4 is an IDP that acts as a potentiator of the Activator Protein-1 (AP-1) transcription factor [128,129]. PAGE4 is phosphorylated by Homeodomain-Interacting Protein Kinase 1 (HIPK1) predominantly at T51 which is critical for its transcriptional activity [130]. However, PAGE4 is also hyperphosphorylated by CDC-Like Kinase2 (CLK2) at multiple S/T residues including T51. Further, while HIPK1 is expressed in both androgen-dependent and androgen-independent PCa cells, CLK2 and PAGE4 are expressed only in androgen-dependent cells. Cell-based reporter assays indicated that PAGE4 interaction with the two kinases leads to opposing functions. Thus, whereas HIPK1-phosphorylated PAGE4 (HIPK1-PAGE4) potentiates c-Jun, CLK2-phosphorylated PAGE4 (CLK2-PAGE4) attenuates c-Jun activity. Consistent with the cellular data, biophysical measurements employing small-angle X-ray scattering, single-molecule fluorescence resonance energy transfer, and multidimensional NMR indicated that HIPK1-PAGE4 exhibits a relatively compact conformational ensemble that binds AP-1, whereas CLK2-PAGE4 is more expanded and resembles a random coil with diminished affinity for AP-1 [106,128].

AP-1 can negatively regulate androgen receptor (AR) activity [131,132], and AR can transcriptionally inhibit CLK2 expression [106]. Furthermore, cells resistant to ADT often have enhanced AR activity (AR protein expression can increase >25 fold) suggesting a positive correlation between ADT resistance and AR activity [133]. These observations combined with the data [106] allowed the construction of a circuit representing the PAGE4/AP-1/AR interactions and the development of a mathematical model that represents the dynamics of this circuit.

**Figure 3:**
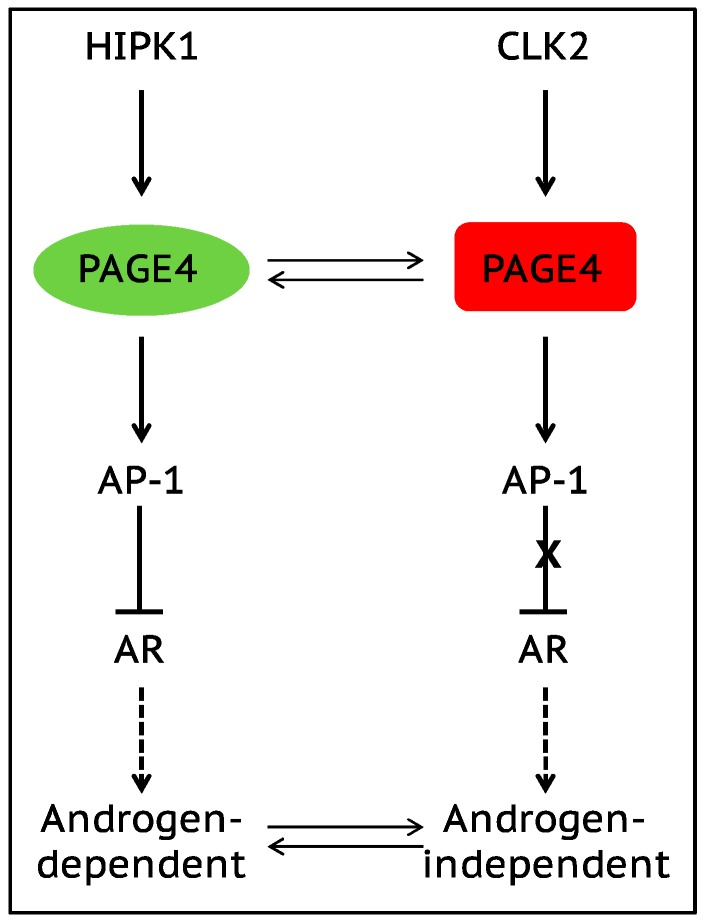
IDP conformational dynamics and phenotypic heterogeneity in prostate cancer cells. The stress-response kinase HIPK1 phosphorylates the IDP PAGE4 resulting in a relatively compact PAGE4 ensemble (HIPK1-PAGE4) that can potentiate AP-1 in androgen-dependent cells. In contrast, the dual-specificity kinase CLK2 hyperphosphorylates PAGE4 leading to a more random-like PAGE4 ensemble (CLK2-PAGE4) that attenuates AP-1 function. Mathematical modeling suggests that the oscillatory dynamics of HIPK1-PAGE4, CLK2-PAGE4, and CLK2 in the circuit enable the cells to transition from an androgen-dependent to an androgen-independent phenotype. This prediction is supported by the experimentally observed heterogeneity in a population of isogenic PCa cells (see [106] for details).

The model predicts that the circuit can display sustained or damped oscillations suggesting that androgen dependence of a cell need not be a fixed state and can vary temporally. Thus, contrary to the prevailing deterministic model that tacitly assumes PCa cells to acquire an androgen-dependent or an independent state (mutually exclusive “binary” model driven by genetic events), cells can enter or exit the androgen-independent state or phenotype (it is reversible) (Figure 3). Even in the case of damped oscillations that eventually settle to one state, the system can revert to displaying sustained oscillations under the effect of biological “noise”. Such noise can originate from multiple sources such as, limited quantities of PAGE4, HIPK1, or CLK2, and/or the conformational dynamics of PAGE4. Furthermore, the model also predicts that the intracellular CLK2, HIPK1-PAGE4, and CLK2-PAGE4 oscillations need not be synchronized across cells. Thus, individual cells in an isogenic population would have varying levels of androgen dependence or independence at a given point in time consequently giving rise to non-genetic phenotypic heterogeneity observed in a seemingly homogenous population of PCa cells [134]. In other words, androgen dependence represents a trait whose values can display a broad distribution across the population. Indeed, this predicted heterogeneity in the levels of HIPK1, CLK2 and PAGE4 is corroborated by quantitative immunohistochemistry and qRT-PCR data [106]. Thus, the model that is developed using the tools of nonlinear dynamics demonstrates how differential phosphorylation of PAGE4 can lead to transitions between androgen-dependent and androgen-independent phenotypes by altering the AP-1/androgen receptor regulatory circuit in PCa cells. Although additional work needs to be done, the study underscores IDPs can stochastically orchestrate phenotypic heterogeneity in PCa due to their conformational dynamics when overexpressed or aberrantly expressed.

## 8. Conclusions and Future Vision

Waddington’s epigenetic landscape initially depicting the differentiation process of stem cells now have been used to understand the phenotypic plasticity in cancer cells. The regulatory network underlying the landscape can give rise to various “attractors”, i.e., “stable states” corresponding to different cell phenotypes, each of which is characterized by a unique gene expression pattern. Emerging insights demonstrate that cancer cells are often behaving as “moving targets” and often find new adaptive ways to resist therapeutic attacks. This search for “cancer attractors” that increase their fitness and/or survival likelihood can be considered akin to “de-canalization”. “Canalization” refers to buffering of biological noise during development, such that cellular phenotypes are stabilized against genetic and/or environmental perturbations, and their variability is decreased [135]. Thus, “de-canalization” would imply supraphysiological plasticity that can make the “valleys” in Waddington’s landscape more shallow (by decreasing the height of the ridge between “valleys”) [136], thereby enabling stochastic sampling of the landscape by cells, hence disrupting the stable cellular phenotypes obtained and maintained in specific niches.

“De-canalization” into “cancer attractors” can be facilitated by gene mutations that rewires the underlying regulatory network. For example, both gain-of-function mutations in proto-oncogenes RAS and MYC and loss-of-function mutations in tumor suppressor genes TP53 and BRCA1 can trigger abnormal cell growth and provoke cancer formation [12]. Once cells enter “cancer attractors”, they acquire high cellular plasticity that allows phenotypic transitions to adjust to the microenvironment. The high plasticity in cancer can be promoted by (a) increased biological noise due to the intrinsic variability in gene expression [137] and the conformational dynamics of intrinsically disordered proteins, such as oncoproteins, reprogramming TFs and EMT-TFs in cancer cells [20,112,113,114]; (b) the changed physiological parameters for cancer cells due to the modified microenvironment [138]. For example, cancer cells usually face hypoxia condition due to their rapid proliferation and the hypoxia condition stabilizes HIF-1. This can then promote cancer cells to acquire a hybrid OXPHOS/glycolysis phenotype that has been shown to be associated with higher metastatic potential as compared with only OXPHOS or glycolysis phenotypes [91]. The high phenotypic plasticity of cancer cells can contribute to metastasis and therapeutic failure.

Recent studies have also highlighted that phenotypic transitions do not have to be cell-autonomous events. Instead, the microenvironment of a cell can often modulate such phenotypic switching, for instance, (a) the lineage commitment of naïve mesenchymal stem cells can be directed by the matrix elasticity and soft matrices generate nerve-like cells, stiff matrices generate muscle-like cells and rigid matrices generate bone-like cells [139], (b) simulated microgravity can dramatically alter the cytoskeletal architecture of MDA-MB-231 cells with consequent effects on proliferation and apoptosis [140], (c) parallel microgrooves on the surface of cell-adhesive substrates can mechanically modulate a cell’s epigenetic state and induce an MET, thereby increasing the efficiency of cellular reprogramming [141], and (d) signals from mammary microenvironment can overrule the ‘terminal commitment’ of a stem cell belonging to a “foreign” tissue [142]. Together, these studies highlight the need to revisit whether a cell is ever “terminally differentiated”, and how much cell-autonomy there is in a cell-fate [143].

## 9. Therapeutic Approach That Promotes “Re-canalization”

Can cells transition from “cancer attractors” back to “normal attractors”, i.e., “re-canalization”? The answer seems to be yes based on some existing data. First, inactivation of the oncogene MYC in hepatocellular carcinoma cells leads to the formation of normal hepatic structures [144]. Second, decreasing the intracellular levels of TCTP (transcriptionally controlled tumor protein) is sufficient to revert the malignancy of MCF7 or T47D cells (breast cancer), U937 cells (histiocytic lymphoma) [145] and v-Src-transformed NIH3T3 cells (fibroblasts) [146], partially through recovering the function of the P53/MDM2 axis [147]. Third, replacement of mitochondria in metastatic triple negative breast cancer cells SUM159 with mitochondria from benign breast cancer cells MCF10A or A1N4 abolish cell migration potential and in vivo tumor formation potential [90]. Forth, modification of the surface integrins of human breast cancer cells in 3-dimensional culture results in a reversion to a normal cell phenotype both morphologically and functionally despite the malignant genome [148]. Therefore, we believe that targeting the sources for phenotypic plasticity in cancer cells, for instance, deactivation of oncoproteins and/or modification of tumor microenvironment can contribute to the “re-canalization”.

Even though it may be difficult to revert cancer cells directly to normal cells, we can still help cancers cells transition out from highly aggressive “attractors”. One possible approach is to perturb factors that help maintain the aggressive “cancer attractors”. For example, knockdown of the phenotypic stability factors OVOL and GRHL2 in H1975 cells can destabilize the hybrid E/M phenotype [54], the “primary bad actors” of metastasis [43,44,46]. Therefore, instead of targeting individual signaling pathways with insufficient knowledge of how they impinge on the epigenetic landscape for each cell, future therapeutic approaches might consider a stepwise approach from the dynamical systems perspective, start with the destabilization of the “cancer attractors”, followed by transitions into “normal attractors”, then deepening the basin of attraction of “normal attractor” to prevent future tumor relapse. As attractive as it may seem, the proposed approach remains to be clarified on the basis of combined modeling and experimental work.
